# Organization of affordance processing in perception-action systems

**DOI:** 10.3389/fnhum.2026.1774789

**Published:** 2026-06-26

**Authors:** Lukasz Przybylski, Gregory Kroliczak

**Affiliations:** 1 Action and Cognition Laboratory, Faculty of Psychology and Cognitive Science, Adam Mickiewicz University, Poznan, Poland; 2 Cognitive Neuroscience Center, Adam Mickiewicz University, Poznan, Poland

**Keywords:** cerebral phenotypes, functional grasp planning, handedness, laterality indices, non-righthanders, phenotypic complexity, tool use pantomimes, visual tool processing

## Abstract

**Introduction:**

Visual tool processing (VTP), functional grasp planning (FGP) and tool use pantomimes (TUP) are typically associated with left-lateralized cerebral mechanisms. Yet, specific relationships between their neural underpinnings and levels of processing involved are largely unknown. We studied whether similar cerebral asymmetries are observed in the three tasks across different handedness groups, and how they link to the three-action system (3AS) model.

**Methods:**

Sixty-two participants (34 non-righthanders) were scanned with fMRI. The same stimuli—12 tools and 12 control objects—were utilized in each task. Whole-brain voxel wise analyses were performed and laterality indices (LIs) calculated in critical regions of interest (ROIs) for the assessment of hemispheric dominance, links to handedness, and global between-task relationships.

**Results:**

Depending on the region, VTP was not left lateralized, unlike FGP and TUP. The contributions of the dorso-dorsal, ventro-dorsal, and ventral stream—in 3AS associated with affordance processing, mechanical, and function knowledge—were contingent on task and phenotypic complexity. LIs for VTP and TUP, and FGP and TUP were significantly correlated. VTP and FGP LIs, unlike the TUP LIs, were linked to handedness. The global between-task relationships were contingent on processing levels.

**Discussion:**

These findings shed new light on affordance encoding, and their ability to automatically invoke neurocognitive mechanisms underlying tool functions and related actions. Future models should consider the mechanisms shared, the levels of processing involved, and their contingence on task complexity, individual variability, and sensorimotor integration, regardless of the involvement of manual responses.

## Introduction

The usage of a concept of an “affordance,” as originally introduced by Gibson (1977, 1979) to denote action possibilities offered by the environment to an organism, has been extended from a seemingly straightforward ‘sensorimotor’ notion to comprehensive, multidimensional research programs encompassing ecological psychology, cognitive neuroscience using electrophysiology or neuroimaging, and the behavioral and/or neuropsychological studies of tool use actions (Warren, 1984; Tucker and Ellis, 1998; Humphreys, 2001; Norman, 2002; Young, 2006; Osiurak et al., 2009; Osiurak et al., 2010; Randerath et al., 2011; Osiurak and Badets, 2016, 2017; cf. Federico et al., 2022; Ras et al., 2022; see also Bluet et al., 2025). The relational character of affordances has been emphasized; namely, they are not merely properties of objects or the more so mental representations, but opportunities for action defined relative to an agent’s capacities and the required sensory-motor integration (Borghi and Riggio, 2015; Sakreida et al., 2016; see also Binkofski and Buccino, 2018; cf. Cisek and Kalaska, 2010; Pezzulo and Cisek, 2016). Efforts to systematize disparate perspectives on affordance processing can be found in the work by Osiurak et al. (2017), who proposed a tripartite architecture of tool-related cognition (a three action-system model: 3AS; cf. 2AS + framework by Buxbaum, 2017), pointing to disparate neural substrates of sensorimotor processes. They involve: hand-centered motor control—putatively contingent on the superior, dorso-dorsal neural stream of processing; tool-centered mechanical knowledge—contingent on the intermediate ventro-dorsal neural stream; and context-driven function knowledge—mediated by the inferior, i.e., ventral stream (Osiurak and Badets, 2016; cf. Buxbaum, 2001; Buxbaum and Kalenine, 2010; Binkofski and Buxbaum, 2013; Goldenberg and Spatt, 2009; Osiurak et al., 2008; Osiurak et al., 2011; see also Goldenberg, 2017; Lesourd et al., 2021). Their framework underscores the conception that affordance processing is an emergent phenomenon arising from the functioning of partially distinct, multiple, semi-independent neural streams, rather than from a single perception- and/or action-dedicated neural pathway, or the more so module (Goodale et al., 2005).

How, and at what stage, the processing within the neural streams underlying affordance and tool-related cognition interacts, and to what extent it is similarly lateralized across individuals with variable organization of functions contingent on hand preference (Kroliczak et al., 2021; Johnstone et al., 2021; Karlsson and Carey, 2024) is an open issue. These critical questions can be addressed by the inclusion in a single project three hypothetically related tasks involving tools: a simple visual/perceptual task, putatively linked to affordance processing *per se*, as well as grasping, and using tools. To this aim, the present study employed the same set of common tools for studying: (1) passive viewing of tools, (2) functional grasp planning, and (3) pantomimed tool use. This enabled us: first, to examine whether affordance processing / recognition is unitary or distributed across multiple neural networks; second, to establish the degree of functional dependence among these networks when stimulus and task parameters are held constant; and third, to identify asymmetries in their lateralization across right- and non-righthanded individuals with disparate organization of skilled actions. Our project was directly inspired by reports postulating the existence of two action streams (Johnson and Grafton, 2003; Rizzolatti and Matelli, 2003), with their later application and extension to tool use (Frey, 2008; Kroliczak and Frey, 2009). Its proposal (Maestro grant # 2011/02/A/HS6/00174) and the first report (Przybylski et al., 2014) preceded seminal review papers on similar topics (Binkofski and Buxbaum, 2013; Osiurak et al., 2017; Buxbaum, 2017; cf. Proietti et al., 2026). Nevertheless, the three tasks that we utilized were hypothesized to invoke neural mechanisms and/or processes that could be easily linked to the neural underpinnings postulated in the 3AS (or 2AS+) model to mediate affordance and other tool-related processing. In the first task—never reported before, and from now on referred to as *Visual Tool Processing* (VTP), primarily ventral and ventro-dorsal contributions were expected. The to-be-invoked neural mechanisms are typically associated with conceptual processing of tools, yet, in the 3AS model are linked to context-driven function knowledge. Our second task (reported earlier in different samples and their subdivisions; i.e., Przybylski and Kroliczak, 2017; Przybylski and Kroliczak, 2023), from now on referred to as *Functional Grasp Planning* (FGP), which could be linked exclusively to hand-centered processing, i.e., the dorso-dorsal pathway in the 3AS model, typically invokes the ventral, as well as ventro-dorsal and dorso-dorsal visual and visuomotor pathways, instead. The third—our main control—task (also reported before in different samples and their subdivisions; Kroliczak et al., 2021; Kroliczak and Przybylski, 2024), from now on referred to as *Tool Use Pantomime* (TUP), which could be linked exclusively to tool-centered processing, i.e., the ventro-dorsal pathway in the 3AS model, typically invokes widespread bilateral neural responses with the left hemisphere advantage observed within the praxis representation network (PRN; Kroliczak and Frey, 2009; but see also Juchtern et al., 2024; Martinez-Addiego et al., 2025). In sum, comparisons of neural activity patterns in the three studied tasks in terms of consistencies in their overall organization and lateralization within critical regions of interest (essential processing nodes or hubs) across righthanded and non-righthanded individuals might be key to refining the neurocognitive models of affordance processing, including earlier theoretical frameworks (Osiurak et al., 2017), and their future extensions (cf. Federico et al., 2025; Osiurak and Federico, 2026; see also Nuzzi et al., 2026).

## Methods

### Participants

Sixty-two participants of this study—28 righthanders (dextrals) and 34 non-righthanders (adextrals) from Poznań universities were tested using fMRI in three tasks during two consecutive sessions. Participants were healthy adults aged 18.7–39.7 years (Mean Age [MA] = 22.6, SD = 3.41). Handedness was determined using the Edinburgh Handedness Inventory (EHI; Oldfield, 1971). Based on criteria used previously (Kroliczak et al., 2021), individuals with EHI_LI_ ≥ +33.3 were classified as righthanders (28 participants; EHI_LI_: M = 91.1, SD = 15.1; MA = 21.7, SD = 1.52), and those with EHI-LI ≤ −33.3 as lefthanders (21 participants; EHI_LI_: M = −76.7, SD = 16.8; MA = 23.4, SD = 3.75). Thirteen participants scored between these cut-offs (mixed-handers EHI_LI_: M = −3.28, SD = 23.8; MA = 23.0, SD = 5.23). Combined, the latter two groups yielded 34 non-right-handed individuals (EHI_LI_: M = −48.6, SD = 41.1; MA = 23.3, SD = 4.30).

### Procedure

#### Visual tool processing (VTP)

**Figure 1:**
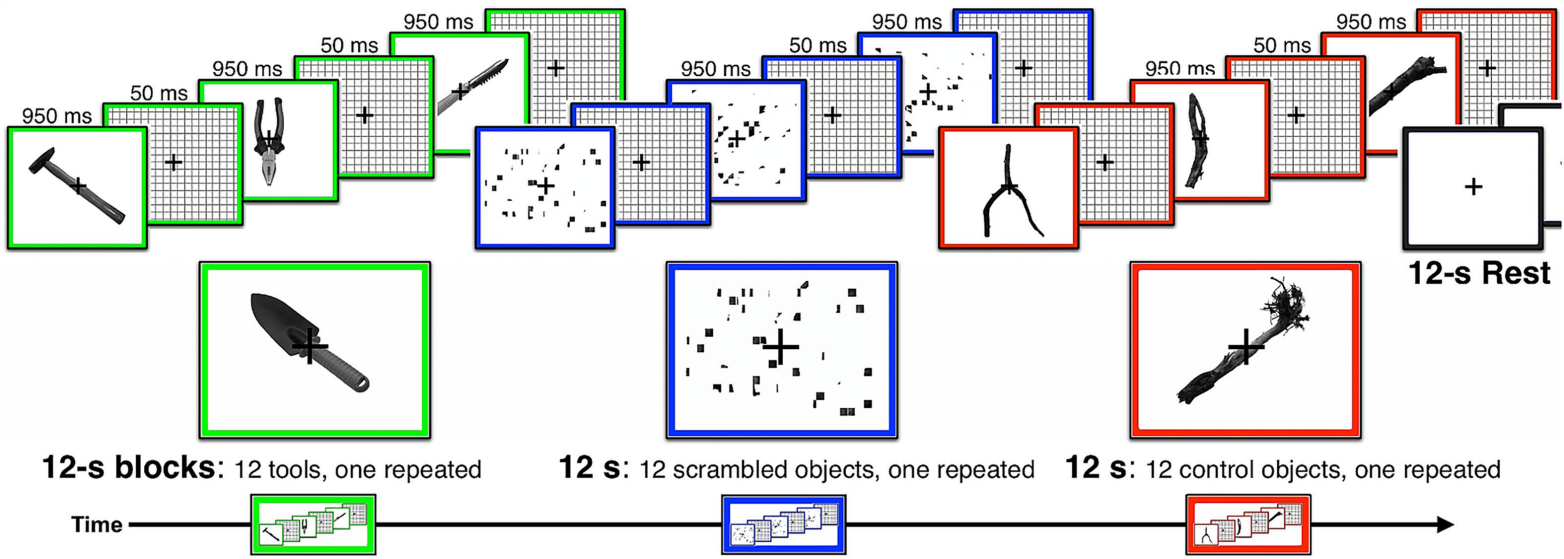
The visual tool processing (VTP) task. The figure shows trial structure and timing, as well as examples of stimuli—tools, scrambled images and control objects—used in VTP. A block design was utilized, where each 12-s block containing only one stimulus type was presented five times, interleaved with six 12-s rest blocks, presented in a pseudorandom order. Twelve images, including one repeated item, were shown for nearly 1 s, followed by a brief mask. There was no motor task, except for a button press for repeated items, controlling for attention.

Twelve images of familiar, common tools (one item repeated) and 12 control object images, such as man-made or natural fragments of wooden sticks shown in Figure 1 (one repeated), along with scrambled counterparts from both categories (also with repeated items), were presented in a pseudorandom sequence within 12-s blocks. Each block type was presented five times, interleaved with six pseudorandomly inserted 12-s rest blocks (see Figure 1). One item repetition in each task block—requiring a button press response, when repetition was noticed—was utilized for controlling participants’ attention. The study involved two runs, administered within two sessions, typically on two following days. In addition to whole-brain voxel-wise contrasts comparing processing of intact tools with control objects, and intact tools with their scrambled versions, laterality indices (LIs) were also calculated (following major guidelines from Vingerhoets et al., 2023). These LIs were then compared with those obtained for the Functional Grasp Planning (FGP) and the visual Tool Use Pantomimes (TUP), enabling an in-depth evaluation of hemispheric involvement across tasks engaging distinct but related components of tool cognition.

#### FGP

The same twelve tool images (as used in VTP) were utilized in the five runs of this task in two scanning sessions. These objects usually have a clearly defined graspable parts. Participants were trained to plan functional grasps of these tools, such that would enable the execution of their typical functions. The same set of 12 control stimuli (i.e., man-made or natural wooden sticks) was also included. The control stimuli did not have clear functional affordances and the planning involved the simplest of possible grasps. The order of the tested hands was counterbalanced. For the most comprehensive description of this task, including all details, see Przybylski and Kroliczak (2017), the report limited to right-handed participants; see also Przybylski and Kroliczak (2023), where results from atypical and typical samples with different handedness status were described, instead.

#### TUP

In the TUP task, utilizing the same stimuli, participants were asked to execute tool use pantomimes, similarly to a procedure described elsewhere (Vingerhoets et al., 2012), but only limited to unimanual responses. As in VTP, there were two runs, typically on two consecutive days; as in FGP, performance with the left and right hand was counterbalanced. The control task involved manual counting of the number of parts in non-tool, control objects described above. Each run comprised five 24-s blocks of tool-use pantomimes and five 24-s blocks of manual part-counting (12 stimuli shown for 2 s in each task), with additional five pseudorandomly interspersed 24-s rest blocks. For the most comprehensive description of this task, including all details, see Kroliczak et al. (2021), and see also Kroliczak et al. (2020). The latter study included a different cohort of participants, who were never tested with the VTP and FGP tasks.

### Data acquisitions, processing and analyses

Neuroimaging data were collected on a Siemens 3 T Trio MRI scanner (Erlangen, Germany) with a 32-channel head coil at the Laboratory of Brain Imaging, Nencki Institute (Warsaw, Poland). Functional BOLD T2*-weighted EPI images had the following parameters: time to repetition (TR) = 2000 ms, time to echo (TE) = 30 ms, flip angle (FA) = 90 degrees, 64 × 64 matrix, 35 axial slices (3.1-mm isotropic voxels). The functional runs encompassed 210 volumes in VTP, 145 volumes in FGP, and 194 volumes in TUP. Structural T1-weighted MP-RAGE scans had the following parameters: TR = 2,530 ms, TE = 3.32 ms, time to inversion (TI) = 1,200 ms, FA = 7°, 256 × 256 matrix, with 176 slices (1-mm isotropic voxels). The alignment of EPI and T1 scans was enhanced with T2-weighted anatomical scans, with TR = 3,200 ms, TE = 402 ms, FA = 120°, 512 × 512 matrix, with 176 slices (0.5 × 0.5 × 1-mm voxels).

Preprocessing and registration were performed in FSL (FEAT v6.0), with standard default settings, and modeling. Individual participant averages utilized fixed effects, with hand-independent estimates obtained by averaging across sessions. Group analyses employed mixed-effects modeling (Beckmann et al., 2003), and the statistical maps were thresholded at least at Z > 3.1 (though typically at half of the maximum Z value, i.e., at Z > 3.8 or higher), with a cluster-wise correction at *p* = 0.05. The multi-modal parcellations from Glasser et al. (2016a) and Glasser et al. (2016b) were included in visualizations of the key outcomes and ROIs using Connectome Workbench v1.4.2. Between-task correlations were visualized using BrainNet (Xia et al., 2013).

**Figure 2:**
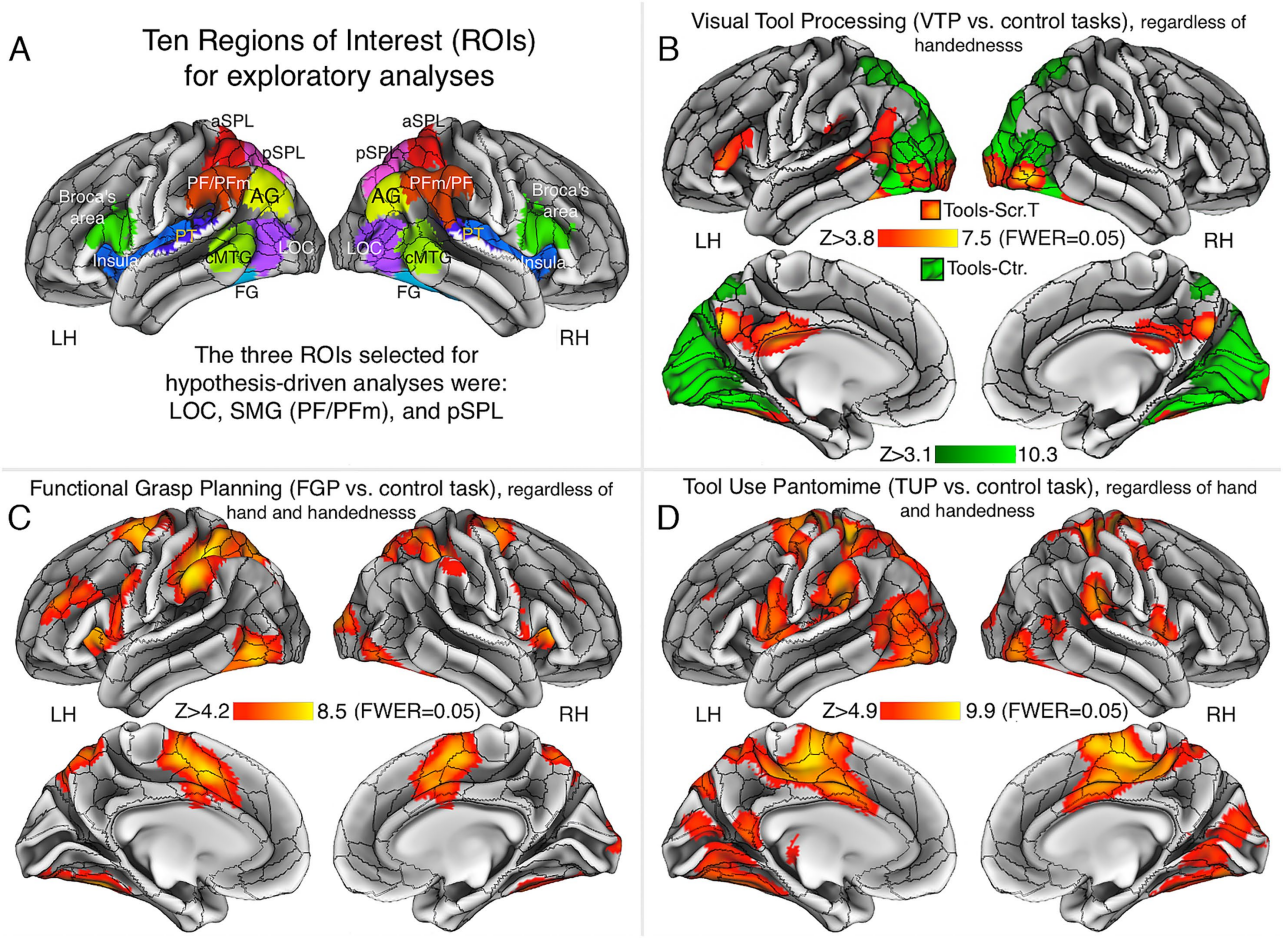
Regions of interest (ROIs) and neural activity related to three main tasks. **(A)** ROIs. Symmetric maps of ten regions constructed from the Juelich Histological and Harvard-Oxford Atlas, or a combination thereof, are shown with their labels and study-relevant descriptions. **(B)** Visual tool processing (VTP). The VTP task contrasted with control objects invoked the medial occipital, ventral and lateral occipito-temporal cortices, as well as superior and medial parietal cortices (shown in shades of green). Note that, in the occipito-temporal areas, neural activity and the associated local peaks are also hidden below red-to-yellow shades. When contrasted with scrambled tools, the VTP task (now shown in red-to-yellow shades) invoked the lateral occipito-temporal, and medial parietal cortices more focally and bilaterally, and the temporo-parieto-occipital cortices, as well as posterior inferior frontal gyrus exclusively on the left. **(C)** Functional grasp planning (FGP). The FGP task invoked greater engagement of the left hemisphere, including occipito-temporal, parietal, as well as frontal regions/areas linked to the praxis representation network (PRN). **(D)** Tool use pantomime (TUP). The TUP task invoked all PRN areas, with greater engagement of the lateral occipito-temporal, and superior occipito-parietal–frontal, as well as retrosplenial cortex on the left, and bilateral medial occipital and medial parieto-frontal cortices.

Regions of interest (ROIs). Three ROIs—i.e., the lateral occipital complex (LOC), the Supramarginal Gyrus (SMG—subdivisions PF/PFm), and the posterior subdivision of the superior parietal lobule (pSPL) were used to investigate the lateralization of processing in all three tasks studied here. Their selection was based on results from previous studies (Kubiak and Kroliczak, 2016; Przybylski and Kroliczak, 2017; Potok et al., 2019; Karlsson and Carey, 2024; Kroliczak and Przybylski, 2024; Pillet et al., 2024), and they were constructed from the Juelich Histological and Harvard-Oxford Atlas, or a combination thereof. Putative relations of the laterality of this processing to the neural underpinnings discussed in the ‘3AS’ model, including SPL for affordance processing, and the contributions of SPL to FGP (including mechanisms related to reaching and grasping), and TUP (engaging hand-centered processes), as well as LOC (engaging context-driven function knowledge) were considered. Non-parametric, Spearman’s correlation coefficients between the three main task-specific LIs, and handedness (from the EHI) were calculated to investigate whether their lateralization within ROIs can be linked to handedness. Further, exploratory between-task LI correlations were also calculated within a set of ten key ROIs, including the aforementioned three main ROIs (Figure 2A; Supplementary Figure S2A) based on knowledge on major processing streams in the brain (i.e., ventral, ventro-dorsal and dorso-dorsal pathways; e.g., Binkofski and Buxbaum, 2013; Rizzolatti and Matelli, 2003; Goodale and Milner, 1992), and the 3AS model by Osiurak et al. (2017). These regions include (1) the occipito-temporal areas (LOC: LO1, LO3, MT/MST, FTG, PH; MTG and Temporo-Parieto-Occipital Junction [TPOJ]: PHT, TE1p, TPOJ1, TPOJ2; the Fusiform Gyrus [FG]: FFC, VVC, TF), typically linked to processing of visual stimuli; as well as subdivisions of the posterior parietal cortex (PPC), i.e., (2) the inferior parietal lobule (IPL, including SMG: PF-PFm, the angular gyrus [AG]: PGs and PGp, partly PGi) and (3) the superior parietal lobule (SPL, including pSPL: IPS1, MIP, VIP, 7PL, but also extending to nearby parcels; and anterior SPL [aSPL]: mainly 7AL, and 7PC), often associated with visuomotor control, as well as manipulation and mechanical knowledge. For completeness, we also included the posterior subdivisions of the inferior frontal gyrus (IFG: subdivisions 44–45, but also 6r and IFJa, i.e., the Broca’s area), and the planum temporale (PT: PBelt, LBelt, RI, and A4), as well as the insula (including AVI, MI, Ig, as well as polymodal and opercular subdivisions), often associated with gesture and language processing (Bidula and Kroliczak, 2015). Most of these areas are responsible for object perception, action planning, and object-directed actions (Goodale et al., 2005).

Consistent with recent guidelines (Vingerhoets et al., 2023), LIs were extracted from symmetrical ROIs. Both LIs for voxel count (VC) and signal intensity (SI) were calculated, but for the sake of simplicity were subsequently averaged (with a rationale provided in Kroliczak et al., 2021), and collapsed across hands/sessions, as described elsewhere (Kroliczak et al., 2021; Kroliczak and Przybylski, 2024). Spearman correlations between LIs were calculated in Jamovi v2.4.1.

## Results

### fMRI analyses

#### VTP

The contrasts shown in Figure 2B revealed extensive bilateral activity in the medial occipital and parietal, as well as ventral and lateral occipito-temporal cortices (VOTC/LOTC), extending dorsally to the superior parietal lobule (SPL), i.e., involving both the ventral and the dorso-dorsal streams of visual processing. These outcomes were particularly pronounced for the Tools–Control objects contrast. In the Tools–Scrambled Tools contrast, more focal and left-sided activity was observed in the caudal middle temporal gyrus (cMTG), temporo-parieto-occipital junction (TPOJ), extending to AG, and opercular parts of the IPL. These areas are linked to conceptual processing of tools and tool-related manual actions. Moreover, a significant cluster was revealed in the mid-to-posterior inferior frontal gyrus (IFG), linked to linguistic processing.

#### FGP

The planning of functional grasps of tools, irrespective of hand and handedness, as depicted in Figure 2C, was associated with extensive neural activity within the praxis representation network (PRN). The IPL, especially the anterior supramarginal gyrus (aSMG), i.e., parcellations PF and PFt, exhibited robust left-hemisphere dominance. So were the middle frontal, ventral premotor and LOTC contributions to this task. Conversely, SPL and dorsal premotor contributions were more bilateral. So were the contributions from the insular and medial frontal cortices. The observed pattern of neural activity involves the ventral, as well as ventro-dorsal and dorso-dorsal visual and visuomotor pathways, responsible for transforming allocentric visual/conceptual (tool-centered) and egocentric motor/visual (hand-centered) information into functional, mechanical and motor knowledge accompanying efficient interactions with tools or their affordances (see Osiurak et al., 2017).

#### TUP

As demonstrated in Figure 2D, a contrast of visually guided tool use pantomimes and a control task of manual counting of object parts revealed a widespread bilateral neural activity, with stronger and more extensive responses observed in the left hemisphere, mainly in PRN. In addition to nearly exclusive contributions from left aSMG (areas PF and PFt), substantially stronger engagement of the ventral stream, including LOTC (comprising areas TPOJ, MT, MST, FST, and PHT, associated with visual object processing and attention), and the dorso-dorsal, superior parietal stream (associated with specification of movement parameters for the pantomiming hand) was observed. There were also greater contributions from the left parietal operculum, and the insular cortex. In contrast, except for the small cluster in the left posterior cingulate and retrosplenial cortex, the extensive medial parieto-frontal activity was largely bilateral. This finding suggests that pantomimed tool use not only involves dorsal sensorimotor processing or integration required for manual reactions triggered by tool affordances but also the ventral perceptual brain pathway associated with visual/conceptual tool affordance processing.

The identified cluster locations, peak coordinates in three main contrasts performed in fMRI voxel-wise analyses, as well as maximum Z-values, and other critical local maxima within identified significant clusters of neural activity are shown in Table 1. Montreal Neurological Institute (MNI) coordinates, and labels from Connectome Workbench, an open-source neuroimaging visualization and analysis platform, were utilized.

**Table 1:** Tasks, contrasts, and MNI coordinates of peak Z values identified in fMRI voxel-wise analyses.

Visual tool processing (VTP)	Tools vs. scrambled tools			MNI coordinates		
Cluster location	Hemisphere	Area in HCP atlas	Z-MAX	X	Y	Z
Occipital Pole	Left	V2	6.87	−26	−102	−4
Lateral Occipital Cortex	Left	LO2	5.86	−46	−84	−8
Angular Gyrus	Left	PGi	4.86	−36	−60	22
Temporo-Parieto-Occipital Junction	Left	TPOJ2	6.32	−54	−64	10
Superior Temporal Sulcus	Left	STSdp	6.22	−48	−42	2
Temporo-Occipital Fusiform Cortex	Left	FFC	6.64	−36	−48	−18
Supramarginal Gyrus, postero-ventral	Left	PSL	5.18	−64	−46	32
Central Opercular Cortex	Left	OP2-3	5.09	−38	−18	22
Parietal Opercular Cortex	Left	PFcm	4.95	−54	−30	22
Precuneus	Left	POS2	6.88	−6	−66	34
Cingulate Gyrus, posterior division	Left	RSC	6.34	−4	−36	26
Inferior Frontal Gyrus, pars triangularis	Left	IFSa	5.34	−50	34	4
Inferior Frontal Gyrus, pars opercularis	Left	44	5.12	−46	20	18
Occipital Pole	Right	V1	7.58	18	−104	0
Lateral Occipital Cortex	Right	V4t	7.6	48	−74	−6
Temporo-Occipital Fusiform Cortex	Right	FFC	6.75	36	−42	−22
Cingulate Gyrus, posterior division	Right	RSC	4.63	6	−44	26

Functional grasp planning (FGP)	Planning grasps of tools vs. control objects			MNI coordinates		
Cluster location	Hemisphere	Area in HCP atlas	Z-MAX	X	Y	Z
Superior Frontal Sulcus, posterior division	Left	6a	8.18	−26	−10	58
Supplementary motor cortex	Left/Right	24dv	7.91	0	2	50
Superior Parietal Lobule	Left	7PC	8.51	−40	−42	50
Supramarginal Gyrus	Left	PF	8.4	−58	−34	36
Supramarginal Gyrus	Left	PFt	7.53	−46	−32	38
Precuneous	Left	7PL	7.16	−14	−68	52
Lateral Occipital Cortex, inferior division	Left	FFC	7.65	−42	−72	−14
Temporal Occipital Fusiform Cortex	Left	VVC	7.18	−32	−56	−18
Lateral Occipital Cortex, inferior division	Left	PH	6.63	−50	−66	0
Middle Frontal Gyrus	Left	46	6.58	−36	42	22
Inferior Frontal Gyrus, pars opercularis	Left	6r	6.9	−54	6	30
Insular Cortex	Left	MI	6.92	−34	20	2
Superior Frontal Sulcus, posterior division	Right	6a	7.82	28	−8	54
Intraparietal Sulcus	Right	AIP	7.12	40	−38	46
Temporal Fusiform Cortex, posterior division	Right	FFC	6.91	34	−42	−24
Occipital Pole	Right	V2	6.89	20	−98	10
Middle Frontal Gyrus	Right	46	5.79	36	42	28
Inferior Frontal Gyrus, pars opercularis	Right	6r	5.18	52	10	4
Frontal Operculum Cortex	Right	FOP5	7.12	32	26	4
Frontal Operculum Cortex	Right	FOP4	5.82	44	18	0

Tool use pantomimes (TUP)	Tool use pantomimes vs. manual part counting			MNI coordinates		
Cluster location	Hemisphere	Area in HCP atlas	Z-MAX	X	Y	Z
Parietal Opercular Cortex	Left	PFcm	8.18	−30	−58	−22
Supramarginal Gyrus	Left	PF	8.09	−60	−36	30
Postcentral Gyrus	Left	1	9.96	−28	−34	64
Postcentral Gyrus	Left	4	9.24	−20	−24	64
Precentral Gyrus	Left	24dd	9.12	6	−12	56
Middle Frontal Gyrus	Left	46	5.96	−38	44	28
Frontal Operculum Cortex	Left	FOP4	5.69	−34	24	14
Inferior Frontal Sulcus	Left	IFSa	5.34	−42	28	12
Lateral Occipital Complex	Left	MST	7.1	−42	−63	9
Fusiform Gyrus	Left	FFC	7.3	−43	−79	−14
Precentral Gyrus	Right	24dd	9.26	2	−18	54
Postcentral Gyrus	Right	4	9.23	26	−26	66
Postcentral Gyrus	Right	3b	8.85	32	−32	62
Frontal Opercular Cortex	Right	FOP4	7.28	38	6	8
Central Opercular Cortex	Right	FOP1	7.14	48	4	6
Parietal Operculum Cortex	Right	PFcm	8.35	44	−30	22
Supramarginal Gyrus, postero-ventral	Right	PSL	8.31	62	−32	24
Supramarginal Gyrus, postero-ventral	Right	PSL	8.21	54	−34	24
Lateral Occipital Complex	Right	MST	6.1	44	−54	8
Temporo-Parieto-Occipital Junction	Right	TPOJ2	6.2	62	−58	8
Cerebellum, V	Left	NA	8.94	−2	−60	−4
Cerebellum, Vermis VI	Right	NA	8.94	2	−66	−18
Putamen	Right	NA	7.08	30	−14	6
Pallidum	Right	NA	6.5	22	8	8

Standard names of cluster locations, the hemisphere they are located in, and their names in the Human Connectome Project (HCP) atlas are described. For each region or area (significant cluster) identified with a given contrast, coordinates of activation peaks in Montreal Neurological Institute (MNI) coordinates are reported. For users of the HCP atlas (Glasser et al., 2016a; Glasser et al., 2016b), the names of multimodal parcels (areas) are provided in addition to terminology/labels from the Harvard-Oxford atlas (Desikan et al., 2006).

### Correlations between LIs from ROIs

As shown in Table 2, despite the fact that all of the studied tasks were tool related, the relationships between LIs (the lateralization of their processing/mechanisms) was partly dissociable. So were the correlations with EHI LIs.

**Table 2:** The correlation matrix between main task LIs within major ROIs, and handedness.

TASK and ROI	VTP LOC	VTP PF/PFm	VTP pSPL	FGP LOC	FGP PF/PFm	FGP pSPL	TUP LOC	TUP PF/PFm	TUP pSPL	EHI LI
VTP LOC	—									
VTP PF/PFm	0.29*	—								
VTP pSPL	0.33**	0.09	—							
FGP LOC	0.31*	0.22	−0.04	—						
FGP PF/PFm	0.10	−0.11	−0.19	0.09	—					
FGP pSPL	0.04	−0.10	0.05	0.16	0.27*	—				
TUP LOC	0.46***	0.09	0.06	0.34**	0.15	0.12	—			
TUP PF/PFm	0.29*	0.07	0.02	0.26*	0.32*	0.26*	0.41***	—		
TUP pSPL	0.07	−0.12	−0.09	0.20	0.41**	0.18	0.28*	0.35**	—	
EHI LI	0.25*	−0.08	0.22	0.17	0.26*	−0.00	0.17	0.11	0.22	—

*p < 0.05, **p < 0.01, ***p < 0.001. Correlation matrix between VTP, FGP, and TUP LIs in three critical regions of interest (ROIs), as well as the EHI LIs for handedness.

Dark grey covers cells showing within task correlations. For significant between task correlations: green covers cells involving LOC and/or PF/PFm, yellow covers cells involving pSPL and PF/PFm, and blue covers cells involving PF/PFm only, i.e., our main regions of interest (ROIs). Finally, light grey covers cells involving significant correlations with handedness.

**Figure 3:**
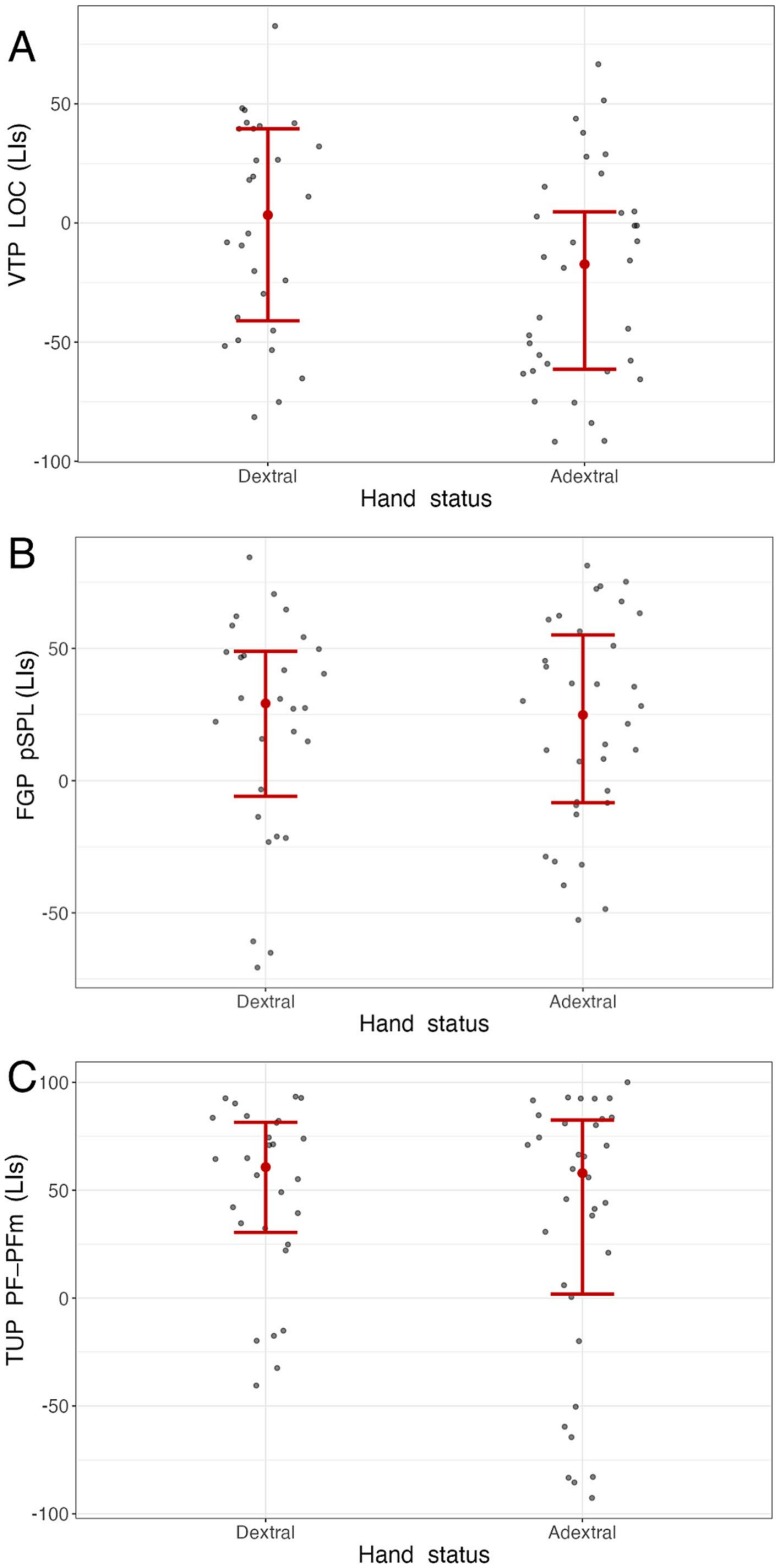
Laterality indices (LIs) for **(A)** visual tool processing (VTP), **(B)** functional grasp planning (FGP), and **(C)** tool use pantomime (TUP) in the lateral occipital complex (LOC), posterior division of the superior parietal lobule (pSPL), and the supramarginal gyrus (subdivisions PF-PFm), respectively. Jittered-density plots for categorical predictors—dextral (right handed), and adextral (non-right handed)—participants were used to visualize the distribution of LIs in each task. Error bars represent the interquartile ranges—25th–75th percentiles—with central tendencies being the median (represented as dots).

**Figure 4:**
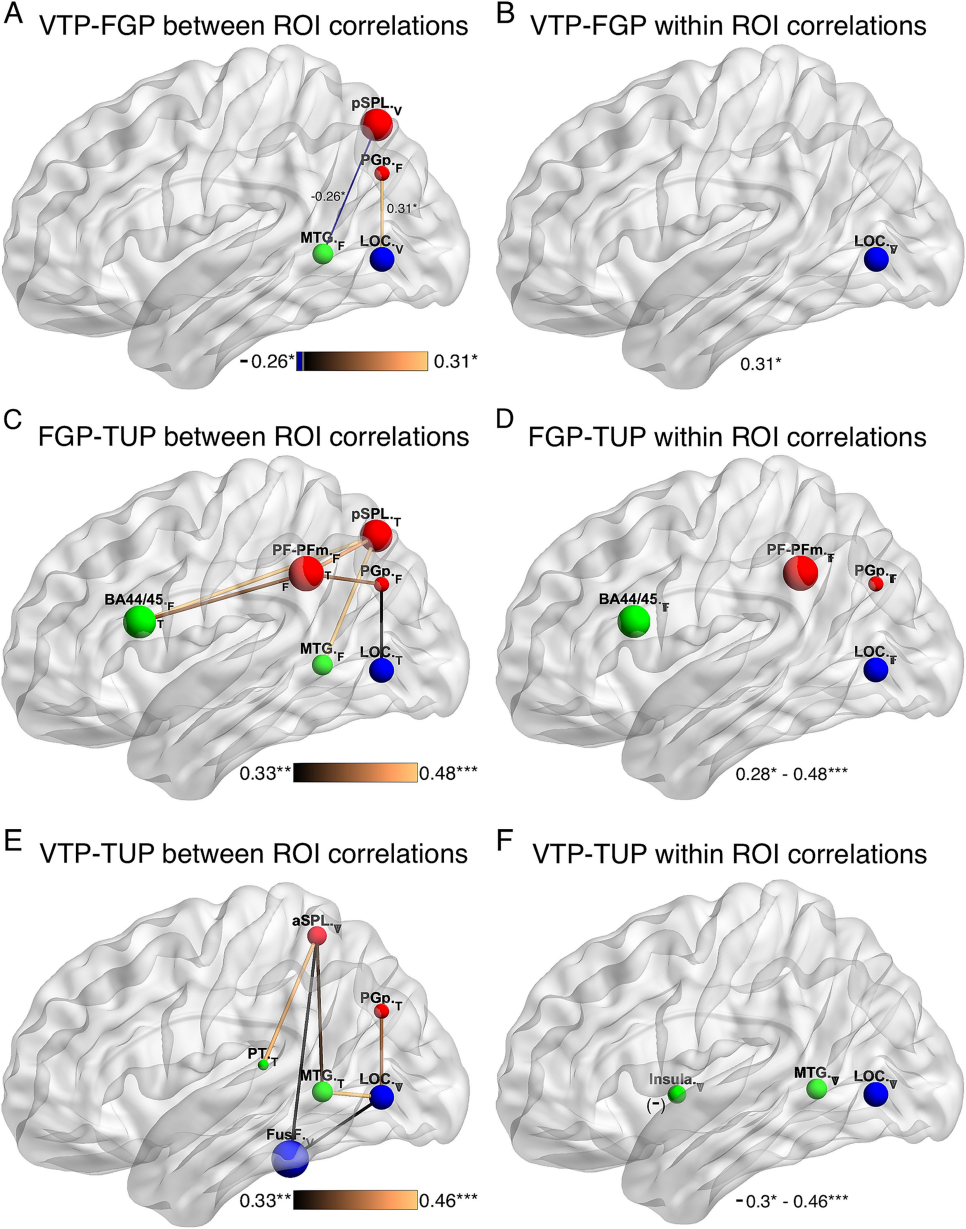
Between-task significant LI correlations identified between and within regions of interest (ROIs). Because we were primarily interested in correlations occurring between the perception-action (or 3AS) systems, significant correlations between ROIs were thresholded at *p* < 0.01 to control for false positives. **Panels A, C,** and **E** show between-ROI correlations, whereas **panels B, D,** and **F** within-ROI correlations. Within ROI correlations were treated as more exploratory, and therefore no correction was applied. We did not have any hypotheses related to negative correlations, and as exploratory, they were also shown in the graphs. All ten ROIs, and all correlations identified are shown in Supplementary material. Subscripts following the ROI (its acronym or name) indicate task: V, visual tool processing; F, functional grasp planning; and T, tool use pantomime. If the ROI shows several correlations—e.g., within and between ROIs—subscripts overlap, forming a symbol: 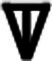 (V&T), 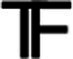 (T&F), and 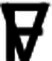 (F&V). The size of the sphere (node) is related to the size of the ROI. The copper colorbars, in combination with thickness of the edges (bars between nodes) show the ranges of significant correlations: the brighter the color/the thicker the edge, the stronger the correlation. In panel **(A)**, the colorbar starts with navy blue shading for negative correlation, also visualized by a very thin navy blue edge.

There was no significant correlation between VTP (in LOC) and FGP (PF/PFm ROI; *ρ* = 0.10, *p* = 0.44), indicating that the lateralization of visual tool processing is not related to the mechanisms underlying functional grasp planning in the supramarginal gyrus. However, a significant positive correlation between VTP (in LOC) and TUP (PF/PFm ROI) was observed (*ρ* = 0.29, *p* < 0.05) indicating that VTP shares some perceptual processing with the tool use pantomime task, at least in the activity of SMG. As shown before (Kroliczak and Przybylski, 2024), FGP and TUP are significantly correlated within SMG, as well (*ρ* = 0.32, *p* < 0.05), which suggests these task share some processing/mechanisms within this ROI. Unexpectedly, EHI LIs are correlated both with VTP (in LOC; *ρ* = 0.25, *p* = 0.05) and FGP (in PF/PFm; *ρ* = 0.26, *p* < 0.05) LIs (yet, VTP in LOC and FGP in PF/PFm and pSPL do not correlate between themselves), suggesting that different aspects of their processing can be linked to handedness. Consistent with an earlier report (Kroliczak et al., 2021), EHI and TUP LIs were not correlated either in any of the main ROIs. Critically, VTP LIs from PF/PFm and pSPL do not correlate with any of the LIs from the other two tasks. The remaining correlations or lack thereof are also shown in Table 2. Notably, the distribution of LIs in all significant correlations from Table 2 is shown in Supplementary Figure S1. Moreover, the distribution of LIs from three different ROIs, contingent on hand status—whether an individual is righthanded/dextral or non-righthanded/adextral—is shown in Figure 3. The outcomes of exploratory analyses of correlations among all ten ROIs utilized in this study are shown in Figure 4.

*VTP-FGP* (Figures 4A,B). The figure reveals weaker and barely existent significant correlations between the two tasks between and within the selected ROIs. They include only one ROI pair (LOC and PGs) for significant positive correlations. If such significant positive correlations indicate the involvement of similar mechanisms, there are few that are common for these two tasks; the other one was identified within LOC (Figure 4B). Only one negative correlation was revealed between MTG and pSPL (Figure 4A; see also Supplementary Table S1; Supplementary Figure S2B), and it involves the dorso-dorsal ROI for the VTP task.

*FGP–TUP* (Figures 4C,D). The figure reveals stronger and numerous significant correlations between the two tasks within the selected ROIs. They include several ROI pairs, in addition to the SMG (PF-PFm) ROI. This finding indicates that FGP and TUP are quite similarly lateralized within the same individuals. This in turn suggests that there are many mechanisms that are common for these two tasks, or their processing interacts at several levels. Indeed, in addition to the dorso-dorsal ROI (pSPL) which in the TUP (subscript T) task is strongly linked to the ventro-dorsal ROIs for the FGP (subscript F) task, the two ventral nodes also show similar lateralization of processing (in one or even two tasks). The remaining outcomes are shown in Supplementary Table S2 and Supplementary Figure S2C.

*VTP-TUP* (Figures 4E,F). The figure also reveals several strong significant correlations between the two tasks, yet within disparate subdivisions of the brain. Except for similar lateralization of aSPL ROI (dorso-dorsal) processing for both tasks, and three more ventral nodes, including PT for tool use pantomime (subscript T), and FusF for visual tool processing (subscript V), the remaining, primarily ventral ROIs—i.e., MTG and LOC—were typically similarly lateralized for both kinds of processing. The remaining outcomes are shown in Supplementary Table S3 and Supplementary Figure S2D.

## Discussion

A fundamental premise of the present study was that affordances are inherently relational, emerging from the interaction between object properties, action goals, and the agent’s sensorimotor capacities. This perspective only partly aligns with the major framework proposed by Osiurak et al. (2017), who differentiated three physical levels of processing and its integration required for tasks involving tools—i.e., affordances (hand centered), mechanical actions (tool centered), and contextual relations (also tool centered). They are based on their neurocognitive underpinnings postulated within the 3AS (three action-system model), i.e., the dorso-dorsal, ventro-dorsal, and ventral streams, respectively. Extending this framework, our findings suggest that these processes (or mechanisms) not only interact but are also distributed across partially dissociable neural systems, an interpretation based on differential lateralization of the underlying fMRI activity, contingent on task demands, and individual phenotypic organization (Figures 2, 3; Supplementary Figure S1).

Across all three tasks—VTP (visual tool processing), FGP (functional grasp planning), and TUP (tool use pantomime)—systematic differences both in hemispheric involvement (lateralization), and disparate configurations of dorsal and ventral regions/modules (hubs or processors) were observed. Moreover, the putative common processes or mechanisms involved in these tasks (as inferred from similar lateralization) ranged from hardly existent (Figures 4A,B) to strong, but involving different substreams, contingent on the task (Figures 4C–F). These patterns do not support a one-to-one mapping to the three neurocognitive systems, and fixed hemispheric specializations. Instead, the division of labor between perceptual/contextual, mechanical, and action-related/motor streams, and subsequent integration of their processing is not complete, and as can be inferred from Figure 4, can be achieved by disparate configurations of hubs and processors in different individuals (as earlier suggested by Price and Friston, 2002).

### The “passive” visual tool processing

The VTP compared to a control task (Figure 2B) evoked bilateral activity in both the ventral system—linked to object recognition and conceptual processing, and the dorso-dorsal system, typically, even if not explicitly, linked to on-line visuo-motor control (Goodale and Milner, 1992; Milner and Goodale, 2008b; Milner and Goodale, 2008a). The latter processing is then automatically potentiated even in tasks involving passive viewing of tools (e.g., Ellis and Tucker, 2000; but see also Squires et al., 2016; Federico and Brandimonte, 2019; Vainio and Ellis, 2020). Critically, the stimuli employed in our task were presented in different orientations (see Figure 1), including two relatively inconvenient ones (if really grasped). While the task did not require explicit processing of object orientation, except for the stimulus repetition in the exact form, where the “yes” response was required, orientation was likely monitored (even if only implicitly), and this was reflected in the engagement of the dorso-dorsal stream (Valyear et al., 2006; Michalowski et al., 2022). This argument aligns with the 3AS assumption that affordances are processed within the dorso-dorsal system. Yet, they are only implicitly processed or invoked, as no relevant manual response (motor control) was required here. This same VTP task substantially engaged functional knowledge from the ventral system, without explicitly invoking mechanical knowledge from the ventro-dorsal system (cf. the 3AS model; Osiurak et al., 2017), despite the fact that object functions were not required to be explicitly monitored, either. Moreover, the requirements for processing of contextual relationships were also minimal in this task, with a focus on simple object features. Yet, somewhat inconsistently with the 3AS model, the ventral stream was substantially engaged (similarly to Valyear et al., 2006; Cavina-Pratesi et al., 2007; see also Chen et al., 2017; Rolls et al., 2026). Interestingly, the VTP contrast with scrambled tools revealed additional left-lateralized contributions from TPOJ, IPL, and IFG—despite the absence of explicit action demands or object naming. Thus, different between- and within-system contributions are revealed here depending on the utilized contrast, wherein tool-related affordances automatically activate both intrinsically action-biased processing, and some conceptual processing/functional knowledge (Wurm and Caramazza, 2022).

### Functional grasp planning

The FGP task (Figure 2C) has been already shown to involve all processing streams or systems regardless of handedness, but was also linked to robust left-hemisphere dominance within core PRN regions (Przybylski and Kroliczak, 2023). Importantly, counter to the VTP task, FGP required explicit processing of object orientation. It was required for proper planning of the positioning of the hand on the graspable parts of the tools, for them to be used according to their typical functions. Unsurprisingly, then, the engagement of the dorso-dorsal stream was inevitable (Valyear et al., 2006; Michalowski et al., 2022; see also Juchtern et al., 2024). Yet, simple egocentric/hand-centered (hand-tool) processing would not suffice for such interactions with tools if they are inconveniently oriented, and the positioning of the graspable vs. functional parts is not properly encoded (Przybylski and Kroliczak, 2017; but see also Bergstrom et al., 2021). Hence, contributions from other systems were required here. Therefore, this same FGP task substantially engaged the ventro-dorsal system, postulated by the 3AS model (Osiurak et al., 2017) to be involved in processing of mechanical knowledge, despite the fact that the requirements for such processing were minimal in our task. So were the requirements for monitoring contextual relationships, as the focus was solely on the tool to be “acted with” (Johnson and Grafton, 2003)—here: grasped accordingly—and recipient objects were not included as stimuli in this task, either. The prominent engagement of IPL subregions, whether or not linked to mechanical knowledge, corroborates that the ventro-dorsal mechanisms/processes extend well beyond simple visuomotor control. Indeed, they possibly engage stored manipulation knowledge, as well (Buxbaum, 2017; see also Binkofski and Buxbaum, 2013; Kroliczak and Ras, 2025), which might then be converted into action plans based on motor-to-mechanical transformations (Ras et al., 2022), even if the latter are only implicitly processed. Function knowledge as such (without broader contexts), is also handy in this task, and indeed supported by visual processing within LOC. This is not surprising as typical tool functions were required to be explicitly included prior to FGP.

### Tool use pantomimes and the 3AS

The TUP task produced the most extensive activity pattern, engaging the dorso-dorsal, ventro-dorsal, and ventral components of the PRN (Kroliczak and Frey, 2009; Przybylski and Kroliczak, 2017), which can be also linked to all three action systems by Osiurak and Badets (2016), Osiurak et al. (2017), and see Figure 2D. The TUP task invoked the dorso-dorsal stream because the stimuli were presented in different orientations, even though orientation was to be disregarded during pantomiming their usage. Substantial engagement of the ventro-dorsal “mechanical knowledge” system (in the 3AS), could be considered surprising because requirements for such processing were also minimal here, and tool manipulation-related movements were explicitly needed. The requirements for monitoring contextual relationships were also minimal, because the focus was on the manual action—manipulation—with putatively held tools and no recipient objects present. Hence, the engagement of IPL in this task corroborates that the ventro-dorsal mechanisms extend beyond invoking mechanical knowledge, if the task requires tool-use pantomimes, instead (Buxbaum, 2017; see also Valyear et al., 2012; Chen et al., 2023). Function knowledge (with any contexts only implicitly invoked), is even more handy in this task, and therefore substantially supported by visual processing within LOC and beyond, i.e., the recruitment of TPOJ in the left hemisphere. All this underscores the critical role of the temporo-parieto-occipital cortex in linking object representations with action schemas.

### Hemispheric asymmetries and handedness

As the correlation analyses in the main ROIs indicate (see Table 2), the lateralization of ventral processing or mechanisms in VTP was not related to the laterality of ventro-dorsal processing or mechanisms invoked in FGP, which is not surprising because VTP did not engage the ventro-dorsal system, and the two tasks focused on processing different aspects of tools. Yet the laterality of ventral encoding in VTP correlated with ventro-dorsal encoding in TUP, even though the tools were also processed differently. Apparently, the two tasks share some common mechanisms, starting from implicit, ventro-dorsal processing of tool manipulation knowledge (mechanical knowledge in 3AS), and the ventral encoding of critical tool features (function knowledge in 3AS). Moreover, FGP clearly shares some mechanisms with TUP (Kroliczak and Przybylski, 2024), even though the latter also shares some mechanisms with VTP, but the former does not. This issue requires further research. Finally, ventral encoding in VTP is weakly, but related to handedness, so is ventro-dorsal encoding in FGP, but not in TUP (consistent with Kroliczak et al., 2021 study on a larger sample). The only reasonable, but tentative, explanation for these discrepancies is the presence of different phenotypes (see Figure 3; see also Supplementary Figure S1), and the associated variability of functional organization in our sample. The VTP-handedness links shown here are somewhat surprising, because Karlsson and Carey (2024) demonstrated that hemispheric asymmetries in tool perception are more closely associated with language dominance rather than handedness.

In the context of ten ROIs, the between-task correlations or lack thereof (Figure 4) can be interpreted as follows. VTP and FGP processing, despite involving the same tool stimuli, is not similarly lateralized within the same individuals (Figure 3; Supplementary Figure S1; Supplementary Table S1). This could result from disparate processing of different, task specific affordances, or just dissociations of the mechanisms involved, at least in terms of their cerebral organization (cf. Karlsson and Carey, 2024). Meanwhile, FGP and TUP, despite invoking disparate key affordances and the related knowledge, i.e., motor and functional vs. manipulation one, show similar lateralization/organization of relevant processing in many ROIs (Supplementary Table S2), including the ones in the ventral system (Bracci et al., 2012). Most of these ROIs are typically associated with the control of skilled actions involving tools (Przybylski and Kroliczak, 2017). Finally, VTP and TUP also show substantial affinities in their processing/lateralization (Supplementary Table S3); yet, it is associated more with functional knowledge, and tool/action semantics, suggesting that these tasks may be also linked by implicit linguistic processing (cf. Thibault et al., 2021).

## Conclusion

While consistent with the 3AS model, with interacting systems for affordance, mechanical and function knowledge processing, the outcomes of our study on simple visual tool processing, functional grasp planning, and tool use pantomimes indicate there is some need for its refinement. The relative contribution of the involved subsystems and the hemispheric specialization for their processing seems contingent on factors such as task complexity, representational demands, and individual variability. Our group data and between-task correlations suggest which mechanisms are shared across the studied tasks and at what level of processing, regardless of hemispheric phenotypes. Our study also sheds new light on neural mechanisms involved in sensorimotor integration, regardless of whether the task involves manual responses or not.

## Data Availability

The datasets presented in this study can be found in online repositories. The names of the repository/repositories and accession number(s) can be found at: DOI: https://doi.org/10.17605/OSF.IO/63HJT.
