# Supplementary material for: Organization of affordance processing in perception-action systems
Source: Front Hum Neurosci. 2026 Jun 26;20:1774789. doi: 10.3389/fnhum.2026.1774789 (PMC13350527; doi:10.3389/fnhum.2026.1774789)
Supplement: Supplementary file 1 [file Data_Sheet_1.pdf]

Supplemental Results (Correlation matrices)

Supplemental Correlation Matrix Table 1 for Fig. 4AB: VTP - FGP Tasks

Correlation Matrix

	VTP_PF_PFm	VTP_PGp	VTP_BA44_45	VTP_MTG	VTP_Fusiform	VTP_Insula	VTP_LOC	VTP_PT	VTP_pSPL	VTP_aSPL	FGP_PF_PFm	FGP_PGp	FGP_BA44_45	FGP_MTG	FGP_Fusiform	FGP_Insula	FGP_LOC	FGP_PT	FGP_pSPL	FGP_aSPL
VTP_PF_PFm	—																			
VTP_PGp	0.23	—																		
VTP_BA44_45	0.47 ***	0.34 **	—																	
VTP_MTG	0.46 ***	0.40 **	0.38 **	—																
VTP_Fusiform	0.10	0.12	0.08	0.36 **	—															
VTP_Insula	0.27 *	0.21	0.38 **	0.17	0.21	—														
VTP_LOC	0.29 *	0.34 **	0.17	0.55 ***	0.38 **	0.08	—													
VTP_PT	0.37 **	0.29 *	0.69 ***	0.29 *	0.14	0.42 ***	0.34 **	—												
VTP_pSPL	0.09	0.18	0.16	0.30 *	-0.03	0.14	0.33 **	0.18	—											
VTP_aSPL	0.09	0.11	0.11	0.16	0.11	-0.00	0.31 *	0.09	0.36 **	—										
FGP_PF_PFm	-0.11	-0.06	-0.01	0.06	0.17	-0.12	0.10	-0.23	-0.19	0.19	—									
FGP_PGp	-0.00	0.19	-0.02	0.01	0.03	-0.05	0.31 *	0.03	0.00	0.09	0.22	—								
FGP_BA44_45	0.06	-0.02	0.13	0.09	0.20	-0.08	0.22	0.14	-0.12	0.10	0.52 ***	0.17	—							
FGP_MTG	0.00	-0.05	-0.02	-0.05	-0.01	0.08	0.06	0.02	-0.26 *	-0.05	0.41 **	0.47 ***	0.36 **	—						
FGP_Fusiform	0.11	0.11	0.06	0.17	0.22	0.09	0.08	-0.02	-0.12	0.10	0.24	0.36 **	0.10	0.14	—					
FGP_Insula	0.01	-0.00	0.08	-0.13	0.12	-0.13	-0.01	0.06	-0.21	0.07	0.37 **	0.24	0.25 *	0.21	0.00	—				
FGP_LOC	0.22	0.06	0.03	0.24	0.07	-0.17	0.31 *	0.10	-0.04	0.14	0.09	0.47 ***	0.16	0.50 ***	0.23	0.09	—			
FGP_PT	-0.01	0.01	0.01	0.13	0.21	0.11	0.08	0.13	-0.00	0.24	0.25 *	0.09	0.11	0.23	0.06	0.12	0.24	—		
FGP_pSPL	-0.10	0.13	0.08	-0.01	-0.02	-0.05	0.04	-0.09	0.05	0.07	0.27 *	0.38 **	0.18	0.30 *	0.20	0.21	0.16	0.02	—	
FGP_aSPL	-0.23	0.20	-0.11	-0.12	-0.17	-0.04	0.00	-0.03	-0.08	0.13	0.40 **	0.15	0.18	0.28 *	0.12	0.17	0.08	0.24	0.40 **	—

Note. * p < .05, ** p < .01, *** p < .001

Supplemental Correlation Matrix Table 2 for Fig. 4CD: FGP - TUP Tasks

Correlation Matrix

	FGP_PF_PFm	FGP_PGp	FGP_BA44_45	FGP_MTG	FGP_Fusiform	FGP_Insula	FGP_LOC	FGP_PT	FGP_pSPL	FGP_aSPL	TUP_PF_PFm	TUP_PGp	TUP_BA44_45	TUP_MTG	TUP_Fusiform	TUP_Insula	TUP_LOC	TUP_PT	TUP_pSPL	TUP_aSPL
FGP_PF_PFm	—																			
FGP_PGp	0.22	—																		
FGP_BA44_45	0.52 ***	0.17	—																	
FGP_MTG	0.41 **	0.47 ***	0.36 **	—																
FGP_Fusiform	0.24	0.36 **	0.10	0.14	—															
FGP_Insula	0.37 **	0.24	0.25 *	0.21	0.00	—														
FGP_LOC	0.09	0.47 ***	0.16	0.50 ***	0.23	0.09	—													
FGP_PT	0.25 *	0.09	0.11	0.23	0.06	0.12	0.24	—												
FGP_pSPL	0.27 *	0.38 **	0.18	0.30 *	0.20	0.21	0.16	0.02	—											
FGP_aSPL	0.40 **	0.15	0.18	0.28 *	0.12	0.17	0.08	0.24	0.40 **	—										
TUP_PF_PFm	0.32 *	0.38 **	0.36 **	0.27 *	0.08	0.11	0.26 *	0.21	0.26 *	0.03	—									
TUP_PGp	0.25	0.28 *	0.30 *	0.03	-0.06	0.12	0.01	0.10	0.17	0.14	0.36 **	—								
TUP_BA44_45	0.39 **	0.12	0.48 ***	0.21	0.06	0.22	0.22	0.16	0.15	0.17	0.35 **	0.40 **	—							
TUP_MTG	0.28 *	0.20	0.24	0.24	0.02	0.11	0.23	0.18	0.05	0.11	0.41 **	0.27 *	0.27 *	—						
TUP_Fusiform	0.16	0.24	0.07	0.25 *	0.13	-0.06	0.03	0.17	0.08	0.01	0.21	0.29 *	0.10	0.34 **	—					
TUP_Insula	0.18	0.06	0.23	0.05	-0.05	-0.09	0.11	0.11	0.11	0.11	0.24	0.18	0.38 **	0.12	0.00	—				
TUP_LOC	0.15	0.33 **	0.23	0.20	-0.01	0.18	0.34 **	0.16	0.12	-0.04	0.41 ***	0.49 ***	0.22	0.40 **	0.43 ***	0.04	—			
TUP_PT	0.15	-0.07	0.15	-0.05	0.20	-0.11	0.10	0.20	0.09	0.02	0.30 *	0.27 *	0.24	0.37 **	0.03	0.45 ***	0.11	—		
TUP_pSPL	0.41 **	0.11	0.43 ***	0.43 ***	-0.04	0.21	0.20	0.09	0.18	0.22	0.35 **	0.41 ***	0.36 **	0.28 *	0.15	-0.02	0.28 *	0.00	—	
TUP_aSPL	0.30 *	-0.04	0.29 *	0.26 *	0.03	0.22	0.31 *	0.24	0.09	0.17	0.20	0.07	0.11	0.26 *	-0.03	0.08	0.19	0.31 *	0.29 *	—

Note. * p < .05, ** p < .01, *** p < .001

Supplemental Correlation Matrix Table 3 for Fig. 4EF: VTP - TUP Tasks

Correlation Matrix

	VTP_PF_PFm	VTP_PGp	VTP_BA44_45	VTP_MTG	VTP_Fusiform	VTP_Insula	VTP_LOC	VTP_PT	VTP_pSPL	VTP_aSPL	TUP_PF_PFm	TUP_PGp	TUP_BA44_45	TUP_MTG	TUP_Fusiform	TUP_Insula	TUP_LOC	TUP_PT	TUP_pSPL	TUP_aSPL
VTP_PF_PFm	—																			
VTP_PGp	0.23	—																		
VTP_BA44_45	0.47 ***	0.34 **	—																	
VTP_MTG	0.46 ***	0.40 **	0.38 **	—																
VTP_Fusiform	0.10	0.12	0.08	0.36 **	—															
VTP_Insula	0.27 *	0.21	0.38 **	0.17	0.21	—														
VTP_LOC	0.29 *	0.34 **	0.17	0.55 ***	0.38 **	0.08	—													
VTP_PT	0.37 **	0.29 *	0.69 ***	0.29 *	0.14	0.42 ***	0.34 **	—												
VTP_pSPL	0.09	0.18	0.16	0.30 *	-0.03	0.14	0.33 **	0.18	—											
VTP_aSPL	0.09	0.11	0.11	0.16	0.11	-0.00	0.31 *	0.09	0.36 **	—										
TUP_PF_PFm	0.07	-0.04	0.05	0.05	0.18	0.01	0.29 *	0.09	0.02	0.10	—									
TUP_PGp	-0.13	0.14	-0.11	0.07	0.20	-0.05	0.38 **	0.02	0.16	0.29 *	0.36 **	—								
TUP_BA44_45	0.07	0.02	0.02	0.20	0.12	-0.25	0.31 *	-0.00	0.03	0.24	0.35 **	0.40 **	—							
TUP_MTG	0.16	0.16	0.23	0.37 **	0.17	-0.01	0.43 ***	0.25 *	0.09	0.35 **	0.41 **	0.27 *	0.27 *	—						
TUP_Fusiform	0.07	-0.07	-0.03	0.21	0.17	0.22	0.26 *	0.08	-0.01	0.02	0.21	0.29 *	0.10	0.34 **	—					
TUP_Insula	-0.00	0.01	-0.13	0.19	0.12	-0.30 *	0.29 *	-0.12	0.09	0.18	0.24	0.18	0.38 **	0.12	0.00	—				
TUP_LOC	0.09	-0.09	0.02	0.20	0.33 **	0.05	0.46 ***	0.18	0.06	0.16	0.41 ***	0.49 ***	0.22	0.40 **	0.43 ***	0.04	—			
TUP_PT	0.16	0.10	0.01	0.25	0.25 *	-0.06	0.25 *	-0.00	0.15	0.42 ***	0.30 *	0.27 *	0.24	0.37 **	0.03	0.45 ***	0.11	—		
TUP_pSPL	-0.12	0.03	0.06	-0.08	0.10	-0.13	0.07	0.01	-0.09	0.06	0.35 **	0.41 ***	0.36 **	0.28 *	0.15	-0.02	0.28 *	0.00	—	
TUP_aSPL	0.16	-0.01	0.06	0.15	0.33 **	-0.01	0.08	0.03	-0.25	0.04	0.20	0.07	0.11	0.26 *	-0.03	0.08	0.19	0.31 *	0.29 *	—

Note. * p < .05, ** p < .01, *** p < .001

References

[1] The jamovi project (2023). *jamovi.* (Version 2.4) [Computer Software]. Retrieved from https://www.jamovi.org.

[2] R Core Team (2022). *R: A Language and environment for statistical computing.* (Version 4.1) [Computer software]. Retrieved from https://cran.r-project.org. (R packages retrieved from CRAN snapshot 2023-04-07).
