# Supplementary figures and images for: Organization of affordance processing in perception-action systems
Source: Front Hum Neurosci. 2026 Jun 26;20:1774789. doi: 10.3389/fnhum.2026.1774789 (PMC13350527; doi:10.3389/fnhum.2026.1774789)

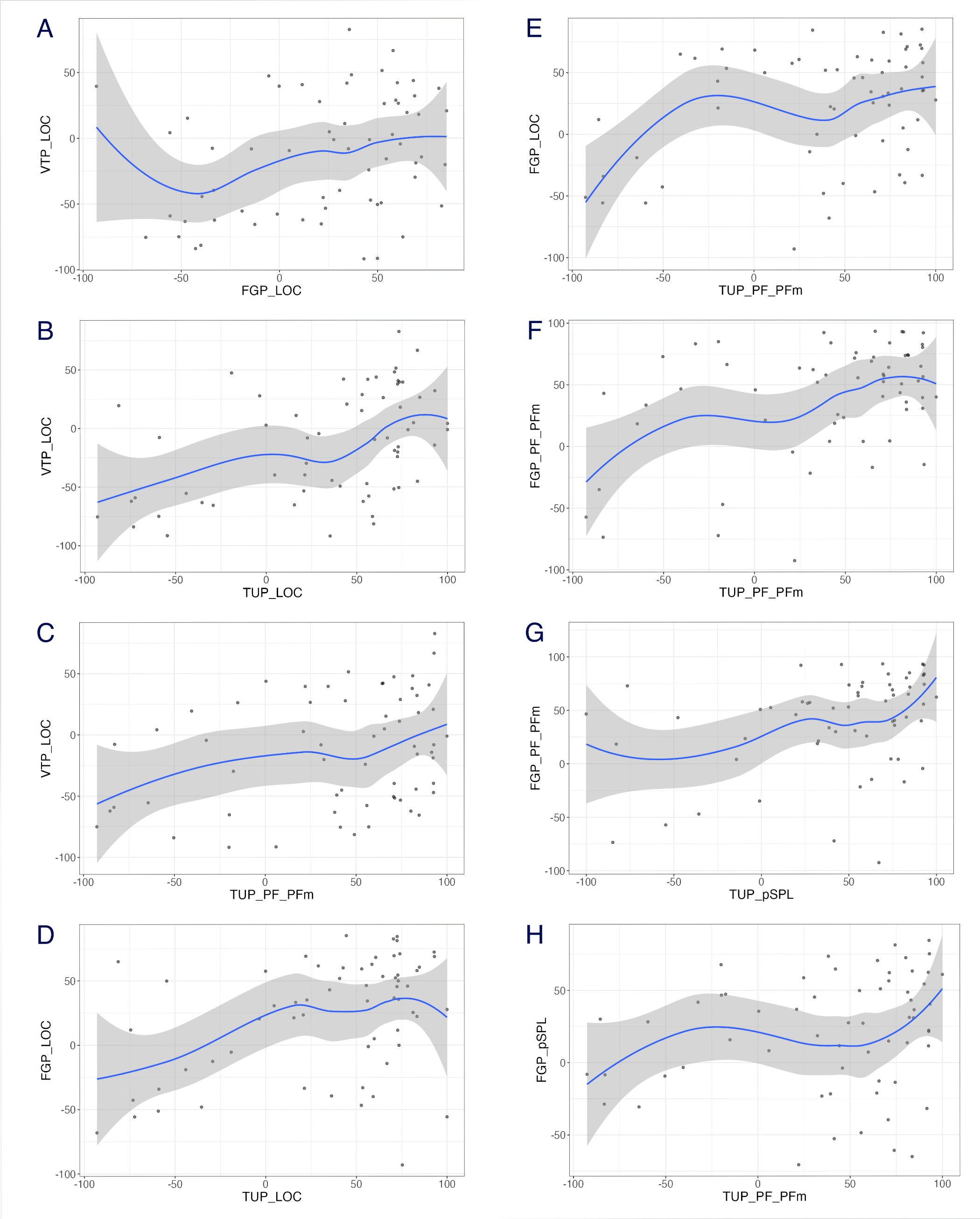

Supplement: Supplementary file 2 [file Image_1.tiff]

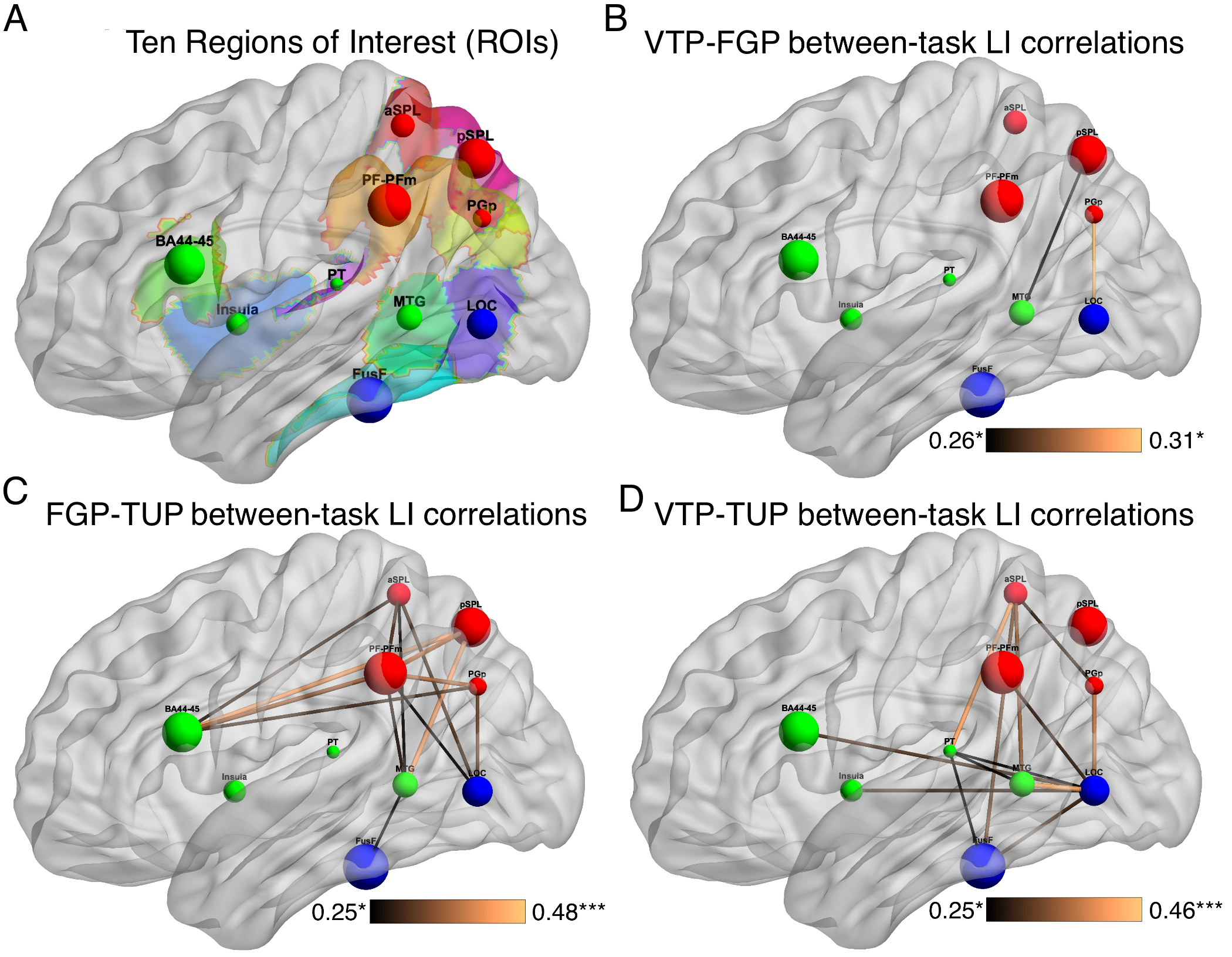

Supplement: Supplementary file 3 [file Image_2.tiff]
